# A New Gene That Shapes Mouse Pigmentation Patterning

**DOI:** 10.1371/journal.pbio.0020016

**Published:** 2004-01-20

Scientists have long known that variation in animal color patterns carry far more than cosmetic significance. Darwin first connected pigmentation with adaptive advantage, noting that male finches with bright red plumage enjoyed greater reproductive success than their drab competitors. Explaining why coloration confers such advantages, however, has proved somewhat easier than showing how it arises. Biologists studying how neighboring regions of the vertebrate body plan develop differences in appearance and form have identified a small number of signaling pathways common to all animals. How and whether these pathways also control the developmental expression and variation of surface attributes like hair color, hair density, and hair length are unclear. By studying an old mouse mutant called *droopy ear*, Gregory Barsh and colleagues show that a member of the well-known family of T-box genes is required for a key pigmentation pattern in mice.

Many vertebrate species—be they fish, bird, or mammal—have a much lighter belly than back. Studies in mice indicate these dorsoventral pigment differences arise from differential expression of the Agouti gene in the ventral and dorsal regions of the developing mouse; Agouti produces a pale yellow color and thus mice with light bellies have Agouti expressed in their ventral but not dorsal region. *Droopy ear* was discovered more than 50 years ago by virtue of its effects on head and ear shape, but it also affects pigmentation patterns; mutant mice have expanded ventral-specific Agouti expression into the dorsal region.

First, Sophie Candille, a graduate student in Barsh's laboratory, searched for the gene that underlies the defect in *droopy ear*. When the researchers homed in on the chromosomal region known to harbor *droopy ear*, they found *Tbx15*—a member of the T-box gene family. T-box genes are found in a wide range of species and play diverse roles during embryonic development. In the *droopy ear* mouse, *Tbx15* carries a mutation that makes the protein nonfunctional. The researchers made certain that *Tbx15* really is the *droopy ear* gene by deleting most of the gene's coding region and showing that this “knocked-out” gene produces the typical *droopy ear* mouse.

The pattern of embryonic *Tbx15* expression—determined by observing messenger RNA transcripts in developing tissues of the head, trunk, and limbs—suggests that early expression of *Tbx15* in the dorsal flank sets coordinates for dorsoventral differences in hair length and pigmentation. Candille et al. demonstrate that the regional pigment differences characteristic of adults is indeed established soon after embryonic *Tbx15* expression. So this boundary in pigmentation is set up very early during development. Interestingly, the early coordinates of the future pigment boundary do not correspond to any other known developmental boundary.

The *Tbx15* pigmentation effects seen in these lab mice, the researchers note, resembles coat variations in other mammals, including German shepherds and an endangered mouse whose lighter dorsal markings once gave it an adaptive advantage on the white sand reefs where it lives (sadly, such markings offer no protection against loss of habitat). T-box genes are also found in humans; mutations in *Tbx1*, *Tbx4*, *Tbx5*, and *Tbx22* can cause developmental abnormalities of the heart, limbs, or of the head and neck. Mutations of human *Tbx15* have not yet been identified, but could contribute to regional differences of pigmentation (in dorsal and ventral surfaces of the limbs, for example) or to development of the head and neck. The identification of *Tbx15* adds a new player to the genes that help pattern the developing embryo—attention now turns to the controls that regulate *Tbx15* and the *Tbx15* targets, which set up the pattern.

**Figure:**
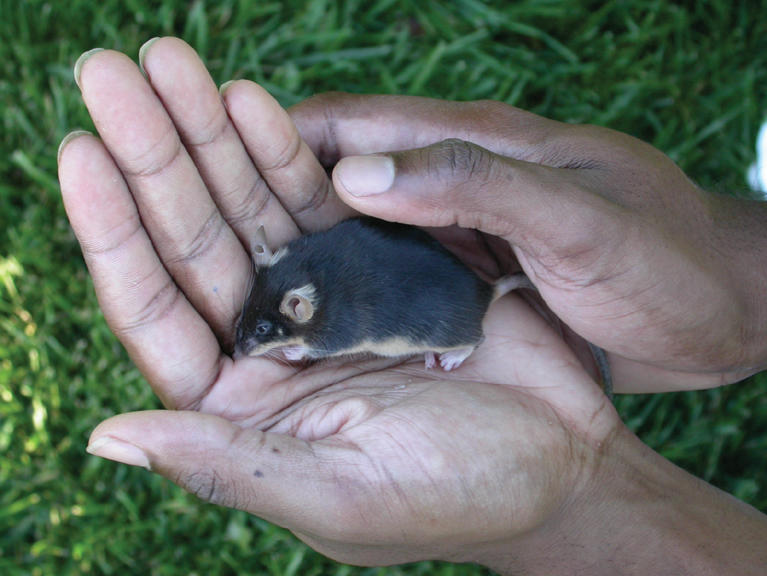
Dorsoventral pigment boundaries in mouse and human

